# Adjusted Empirical Likelihood Method in the Presence of Nuisance Parameters with Application to the Sharpe Ratio

**DOI:** 10.3390/e20050316

**Published:** 2018-04-25

**Authors:** Yuejiao Fu, Hangjing Wang, Augustine Wong

**Affiliations:** Department of Mathematics and Statistics, York University, 4700 Keele Street, Toronto, ON M3J 1P3, Canada

**Keywords:** adjusted empirical likelihood, coverage probability, nonparametric, nuisance parameter, Sharpe ratio

## Abstract

The Sharpe ratio is a widely used risk-adjusted performance measurement in economics and finance. Most of the known statistical inferential methods devoted to the Sharpe ratio are based on the assumption that the data are normally distributed. In this article, without making any distributional assumption on the data, we develop the adjusted empirical likelihood method to obtain inference for a parameter of interest in the presence of nuisance parameters. We show that the log adjusted empirical likelihood ratio statistic is asymptotically distributed as the chi-square distribution. The proposed method is applied to obtain inference for the Sharpe ratio. Simulation results illustrate that the proposed method is comparable to Jobson and Korkie’s method (1981) and outperforms the empirical likelihood method when the data are from a symmetric distribution. In addition, when the data are from a skewed distribution, the proposed method significantly outperforms all other existing methods. A real-data example is analyzed to exemplify the application of the proposed method.

## 1. Introduction

In financial economics, Sharpe ratio, defined in [1], provides a measure of a fund’s excess returns relative to its volatility. Let μ be an expected return of an asset, and σ be the corresponding standard deviation. The Sharpe ratio is defined as

$$sr=\frac{\mu -R_{f}}{\sigma },$$

where $R_{f}$ is a known risk-free rate of return. Note that the larger the Sharpe ratio is, the more return the investor is getting per unit of risk. It is the standard convention in economics and finance research to report the Sharpe ratio. Therefore, the Sharpe ratio is very well studied as a measure of the mutual fund performance in the financial economic areas such as the portfolio analysis, the pricing of capital asset under conditions of risk and the general behavior of stock market prices. The popularity of the Sharpe ratio in financial economics is not only from its simplicity; the study of the Sharpe ratio will also directly result in deeper understandings in portfolio selections. Assuming that the asset returns are all normally distributed, Sharpe [1] showed that picking an asset with the largest Sharpe ratio is equivalent to finding a solution of the investor’s expected utility problem.

Under the normality assumption, Jobson and Korkie [2] proposed a parametric test for the Sharpe ratio, which is a very popular inferential method in economics and finance. However, as shown by many researchers [3,4,5,6], it is very common for the actual returns of the investments, such as the hedge funds, to have a skewed distribution. When the normality assumption of the investment returns is violated, the commonly used approximate distributions of the Sharpe ratio which are developed under the normality assumption become problematic. Model mis-specification is a big concern for all parametric approaches since a misspecified model may lead to biased results. Since the Sharpe ratio is only involved in the first two moments of the data, one of the themes attempting to resolve the problem is to consider higher order moments. There was abundant literature along this line of research such as [7,8,9,10] and references therein.

Another line of research to the problem is to use the nonparametric approach. In this article, we adopt the empirical likelihood (EL) method. Empirical likelihood-type method was first used by Thomas and Grunkemeier [11] to study the survival probabilities estimated by the Kaplan–Meier curve. Owen [12,13] formalized the EL as a unified inference method under more general settings. The EL-based confidence region has several beneficial properties: it does not impose prior constraints on region shape, is transformation invariant and Bartlett correctable [14]. Qin and Lawless [15] applied the EL to inference on parameters that are generated from estimating equations. When the sample size is small and/or the dimension of the estimating equations is high, the EL approach can be hindered by an empty set problem and under-coverage problem. In order to resolve the empty set problem and improve the coverage probability of the statistical tests of the ordinary EL methods, Chen et al. [16] proposed the adjusted empirical likelihood (AEL) method by adding one artificial point into the data set. However, only problems without nuisance parameters were considered in [16]. In this article, we focus on the AEL method with nuisance parameters in addition to the parameter of primary interest. We develop the asymptotic theory of the AEL method when nuisance parameters exist, and demonstrate the use of the AEL method in the application of the Sharpe ratio. Our simulation studies show that the proposed approach provides a beneficial robust alternative to the inference of the Sharpe ratio. The proposed AEL method is comparable to Jobson and Korkie’s method [2] and outperforms the EL method when the data are from a symmetric distribution, while for data generating from a skewed distribution, the proposed method outperforms all other existing methods, especially for small sample sizes. The AEL method preserves the advantage of the EL method: the shape of confidence region based on the AEL ratio reflects the observed data set, while the confidence region based on other methods (excluding EL) is always symmetric about the point estimator. Therefore, the AEL approach allows the data to speak for themselves, and is robust against model mis-specification.

The rest of the article is organized as follows. A brief introduction to the EL and AEL methodologies is given in Section 2. In Section 3, we study the asymptotic property of the AEL method with nuisance parameters. In Section 4, simulation studies are conducted to investigate the precision of the coverage probabilities in the context of the Sharpe ratio. In Section 5, a real-data example is analyzed to illustrate the application of the proposed method. Some concluding remarks are given in Section 6. The technical details are presented in the Appendix.

## 2. Review of the Empirical Likelihood and the Adjusted Empirical Likelihood Methods

Let $X_{1},X_{2},\ldots ,X_{n}\in R^{d}$ be the independent and identically distributed random vectors following distribution *F* with mean μ and a nonsingular covariance matrix. The corresponding observed values are denoted by $x_{1},x_{2},\ldots ,x_{n}$. The EL function for the population distribution *F* is given by

$$L\left(F\right)=\prod_{i=1}^{n}F\left(\left\{x_{i}\right\}\right),$$

where $F(\left\{x_{i}\right\})$ is the probability of observing the value $x_{i}$ in a sample from *F*. Denote $p_{i}=F\left(\left\{x_{i}\right\}\right)$. The EL function can also be written as

(1) $$L\left(F\right)=\prod_{i=1}^{n}p_{i}.$$

Clearly , we have $0\leq p_{i}\leq 1$ and $\sum_{i=1}^{n}p_{i}=1$. Suppose that the goal is to construct a confidence region for the mean μ. The profile EL function of μ is defined to be

$$L_{EL}\left(\mu \right)=\sup\left\{\prod_{i=1}^{n}p_{i}:\;p_{i}\geq 0,i=1,\ldots ,n;\;\sum_{i=1}^{n}p_{i}=1;\;\sum_{i=1}^{n}p_{i}x_{i}=\mu \right\}.$$

Qin and Lawless [15] showed that extra information in the form of a set of estimating equations can be used to improve the maximum empirical likelihood estimators (MELE) and the EL ratio confidence intervals. Suppose a *k* dimensional parameter θ is associated with *F* via a vector g(x,θ) of r≥k functionally independent unbiased estimating functions. Then for each j=1,2,…,r, we have an estimating equation $E_{F}\left\{g_{j}\left(x,\theta \right)\right\}=0$, which can be written in the vector form as $E_{F}\left\{g\left(x,\theta \right)\right\}=0$. The profile EL function of θ is

(2) $$L_{EL}\left(\theta \right)=\sup\left\{\prod_{i=1}^{n}p_{i}:\;p_{i}\geq 0,i=1,\ldots ,n;\;\sum_{i=1}^{n}p_{i}=1;\;\sum_{i=1}^{n}p_{i}g\left(x_{i},\theta \right)=0\right\},$$

and hence, the profile log-EL function is

(3) $$l_{EL}\left(\theta \right)=\sup\left\{\sum_{i=1}^{n}\log p_{i}:\;p_{i}\geq 0,i=1,\ldots ,n;\;\sum_{i=1}^{n}p_{i}=1;\;\sum_{i=1}^{n}p_{i}g\left(x_{i},\theta \right)=0\right\}.$$

The constrained optimization problem in (3) can be solved by applying the method of Lagrange multipliers. Let λ and $t={\left(t_{1},\ldots ,t_{r}\right)}^{\tau }$ be Lagrange multipliers and define

(4) $$H=\sum_{i}\log p_{i}+\lambda \left(1-\sum_{i}p_{i}\right)-nt^{\tau }\sum_{i}p_{i}g\left(x_{i},\theta \right).$$

Then maximizing (3) is equivalent to maximizing *H* unconditionally. Setting the first partial derivative of (4) with respect to $p_{i}$ equal to 0, we have

$$\frac{\partial H}{\partial p_{i}}=\frac{1}{p_{i}}-\lambda -nt^{\tau }g\left(x_{i},\theta \right)=0,$$

$$\sum_{i=1}^{n}p_{i}\frac{\partial H}{\partial p_{i}}=n-\lambda =0\;\;\;\;\Rightarrow \;\;\;\;\lambda =n$$

and

$${\hat{p}}_{i}=\frac{1}{n[1+t^{\tau }g\left(x_{i},\theta \right)]}\;\;,$$

where *t* can be expressed as a function of θ by solving the following equations

(5) $$\sum_{i=1}^{n}{\hat{p}}_{i}g\left(x_{i},\theta \right)=0.$$

Now the profile log-EL function can be written as

(6) $$l_{EL}\left(\theta \right)=-\sum_{i=1}^{n}\log\left[1+t^{\tau }g\left(x_{i},\theta \right)\right]-n\log n.$$

Note that (5) can be rewritten as

(7) $$\sum_{i=1}^{n}\frac{g(x_{i},\theta )}{1+t^{\tau }g\left(x_{i},\theta \right)}=0.$$

Now maximizing (3) has been transformed into an equivalence of solving (7) for the Lagrange multiplier *t*. In practice, this is achieved by numerical methods. One such algorithm devoted to this end can be found in [16]. A necessary and sufficient condition for the existence of a solution $\tilde{t}=\tilde{t}\left(\theta \right)$ in (7) is that 0 must be an inner point of the convex hull expanded by $\left\{g\left(x_{i},\theta \right),i=1,2,\ldots ,n\right\}$.

Qin and Lawless [15] further showed that under some regularity conditions, the EL ratio statistic $W_{0}\left(\theta_{0}\right)=2\left[l_{EL}\left(\tilde{\theta }\right)-l_{EL}\left(\theta_{0}\right)\right]$ converges to $\chi_{k}^{2}$ in distribution as the sample size *n* approaches infinity. This result is the foundation for hypothesis test on θ and can be used to construct an approximate 100(1−α)% confidence region of θ,

$$I_{EL}=\left\{\mu :W_{0}\left(\theta \right)\leq \chi_{k}^{2}\left(1-\alpha \right)\right\},$$

where $\chi_{k}^{2}\left(1-\alpha \right)$ is the 100(1−α)% quantile of the $\chi_{k}^{2}$ distribution, and α is a pre-specified significance level.

Under mild conditions, the convex hull of $\left\{g\left(x_{i},\theta \right),\;\;i=1,2,\ldots ,n\right\}$ contains 0 as its inner point with probability 1 as n→∞. However, if θ is not close to the true parameter $\theta_{0}$ or when the sample size *n* is small, the convex hull is not guaranteed to contain 0. Thus, there is a nonzero probability that the solution to (7) does not exist. It results computational issues when solving the constrained optimization problem in the definition of the EL function. This is known as the empty set problem or the convex hull problem in the EL literature.

In order to resolve the convex hull problem, Chen et al. [16] proposed the AEL method by adding one artificial point into the data set. Denote

$$g_{i}=g_{i}\left(\theta \right)=g\left(x_{i},\theta \right)$$

and

$${\bar{g}}_{n}={\bar{g}}_{n}\left(\theta \right)=\frac{1}{n}\sum_{i=1}^{n}g_{i}.$$

Let $a_{n}=o\left(n\right)$ be a given positive constant. Define a new point by

$$g_{n+1}=g_{n+1}\left(\theta \right)=-\frac{a_{n}}{n}\sum_{i=1}^{n}g_{i}=-a_{n}{\bar{g}}_{n}.$$

Similar to (2), the profile log-AEL function if defined as

$$l_{AEL}\left(\theta \right)=\sup\left\{\sum_{i=1}^{n+1}\log\left[\left(n+1\right)p_{i}\right]:\;p_{i}\geq 0,i=1,\ldots ,n+1;\;\sum_{i=1}^{n+1}p_{i}=1;\;\sum_{i=1}^{n+1}p_{i}g_{i}=0\right\},$$

and we have

(8) $$l_{AEL}\left(\theta \right)=-\sum_{i=1}^{n+1}\log\left[1+t^{\tau }g\left(x_{i},\theta \right)\right],$$

where *t* satisfies

(9) $$\sum_{i=1}^{n+1}\frac{g(x_{i},\theta )}{1+t^{\tau }g\left(x_{i},\theta \right)}=0.$$

The introduction of $g_{n+1}$ guarantees a solution for *t* in (7). Let the maximum AEL estimator $\tilde{\theta }$ be the maximizer of $l_{AEL}\left(\theta \right)$. Under mild regularity conditions, the AEL ratio statistic $W\left(\theta_{0}\right)=2\left[l_{AEL}\left(\tilde{\theta }\right)-l_{AEL}\left(\theta_{0}\right)\right]$ converges to $\chi_{k}^{2}$ in distribution as the sample size *n* approaches infinity. Chen et al. [16] showed that the statistical tests based on the AEL method give better coverage probabilities than those obtained by the original EL method.

In this article, we propose using the AEL method to conduct inference on the Sharpe ratio. Suppose the data is from a population with mean μ and variance $\sigma^{2}$. Without loss of generality, for the rest of this article, define the Sharpe ratio of the population as

$$sr=\frac{\mu }{\sigma }.$$

In this case, the parameter vector is $\theta =(\mu ,\sigma^{2})$, and the parameter of interest is sr. The set of estimating functions can either be

(10) $$X-\mu \;\;\;\;\mathrm{and}\;\;\;\;{\left(X-\mu \right)}^{2}-{\left(\frac{\mu }{sr}\right)}^{2}$$

or

(11) $$X-\sigma \left(sr\right)\;\;\;\;\mathrm{and}\;\;\;\;{\left(X-\sigma \left(sr\right)\right)}^{2}-\sigma^{2},$$

which has μ or σ as the nuisance parameter, respectively. Chen et al. [16] discussed the AEL-based inference without nuisance parameters. Building upon [15,16], we develop the convergence theorem for the AEL with nuisance parameters as shown in the next section.

## 3. The Adjusted Empirical Likelihood Method in the Presence of Nuisance Parameters

Suppose a *k* dimensional parameter $\theta =(\theta_{1},\theta_{2})$ consists a *q* dimensional parameter of interest $\theta_{1}$ as well as a (k−q) dimensional nuisance parameter $\theta_{2}$. The goal is to test $H_{0}:\theta_{1}=\theta_{1}^{0}$ for some given $\theta_{1}^{0}$. In order to obtain inference for $\theta_{1}$ using the AEL method, the asymptotic results in [16] need to be reconstructed and extended to the situation with nuisance parameters.

First, we develop a lemma about positive definite matrices. If a matrix *M* is positive semidefinite, we denote it by M≥0; if *M* is positive definite, we write M>0. For any matrices *G* and *H*, let G≥H denote that G−H is positive semidefinite, and let G>H denote that G−H is positive definite.

**Lemma** **1.** *Let M be a k×k symmetric positive definite block matrix of the form*

$$M=\left(\begin{array}{cc} A & B\\ B^{\tau } & C \end{array}\right),$$

*where A is a q×q matrix, B is a q×(k−q) matrix, and C is a (k−q)×(k−q) matrix. Then C is positive definite and*

$${\left(\begin{array}{cc} A & B\\ B^{\tau } & C \end{array}\right)}^{-1}\geq \left(\begin{array}{cc} 0 & 0\\ 0 & C^{-1} \end{array}\right).$$

The proof of the above lemma is given in Appendix. In order to prove the main theorem, we also need the following two results about idempotent matrices. The proof of these two results can be found in [17] (pp. 186–187).

**Result** **1.** *A necessary and sufficient condition that $Y^{\prime }AY$ has a $\chi^{2}$ distribution is that A is idempotent, that is, $A^{2}=A$, in which case the degrees of freedom of $\chi^{2}$ is rank A = trace A.*

**Result** **2.** *If A, B, A−B are matrices of non-negative quadratic forms and *A* and *B* are idempotent, then A−B is also idempotent.*

Based on Lemma (1) and the above two results, we have the following theorem which gives the asymptotic properties of the AEL ratio test statistic. The theorem is a nonparametric analogue of the theorem in [18] on the asymptotic distribution of the likelihood ratio. The difference is that Wilks’ theorem is based on parametric likelihood and ours is based on the adjusted empirical likelihood. Moreover, it takes into consideration nuisance parameters. We follow the idea of profiling out nuisance parameters (Corollary 5 in [15] and Corollary 1 in [19]) to perform the AEL ratio test. The proof of the theorem is provided in Appendix.

**Theorem** **1.** *Let $\theta^{\tau }={\left(\theta_{1},\theta_{2}\right)}^{\tau }$, where $\theta_{1}$ and $\theta_{2}$ are q×1 and (k−q)×1 vectors, respectively. For $H_{0}:\theta_{1}=\theta_{1}^{0}$, the profile AEL ratio test statistic is*

$$W\left(\theta_{1}^{0}\right)=2\left[l_{AEL}\left({\tilde{\theta }}_{1},{\tilde{\theta }}_{2}\right)-l_{AEL}\left(\theta_{1}^{0},{\tilde{\theta }}_{2}^{0}\right)\right],$$

*where ${\tilde{\theta }}^{\tau }={\left({\tilde{\theta }}_{1},{\tilde{\theta }}_{2}\right)}^{\tau }$ maximizes $l_{AEL}\left(\theta \right)=l_{AEL}\left(\theta_{1},\theta_{2}\right)$, and ${\tilde{\theta }}_{2}^{0}$ maximizes $l_{AEL}\left(\theta_{1}^{0},\theta_{2}\right)$ with respect to $\theta_{2}$. Under $H_{0}$, $W\left(\theta_{1}^{0}\right)\;\overset{d}{\to }\;\chi_{q}^{2}$ as n→∞.*

It is worth noticing that Theorem 1 holds true as long as $a_{n}=o_{p}\left(n\right)$. In application, $a_{n}$ with higher orders is usually not recommended, since the AEL ratios are decreasing functions of the adjustment level $a_{n}$ [20]. As suggested by [16], we set $a_{n}=\frac{1}{2}\log n$ for all of the simulations and applications if not otherwise specified.

Since in Theorem 1 , $\theta_{1}$ is the parameter of interest and $\theta_{2}$ is considered as the nuisance parameter. We can apply the theorem to the Sharpe ratio by setting $\theta_{1}=sr$ along with $\theta_{2}=\mu$ or $\theta_{2}=\sigma^{2}$. Therefore, the AEL ratio statistic under the null hypothesis $H_{0}:sr=sr_{0}$ can be either

(12) $$W\left(sr_{0}\right)=2\left[l_{AEL}\left(\tilde{sr},\tilde{\mu }\right)-l_{AEL}\left(sr_{0},{\tilde{\mu }}_{0}\right)\right],$$

or

(13) $$W\left(sr_{0}\right)=2\left[l_{AEL}\left(\tilde{sr},{\tilde{\sigma }}^{2}\right)-l_{AEL}\left(sr_{0},{\tilde{\sigma }}_{0}^{2}\right)\right].$$

Our simulation shows that using (12) or (13) as the AEL ratio statistic does not make any significant difference in the inference of sr.

## 4. Simulation Study

In order to evaluate the accuracy of the asymptotic chi-square calibration of the AEL method, we choose the coverage probability as an indicator throughout this section. For some fixed sample size *n* and $sr_{0}$, suppose we have run the simulation *m* times and *s* of the simulated $W(sr_{0})$ are less than the 1−α quantile of $\chi_{1}^{2}$ for some given α. Then the coverage probability is defined to be s/m, which is compared with the nominal value 1−α. When *m* is large, if the coverage probability s/m is close to 1−α, then the level α test for sr will tend to give good performance and $\chi_{1}^{2}$ is considered an acceptable reference distribution for $W(sr_{0})$ at sample size *n*.

We compare the coverage probability of the proposed method with other methods for sample sizes n=20,50,200,500 at nominal values 1−α=0.9,0.95. Each coverage probability is obtained from m=5000 simulations. The data are generated from the normal distribution with mean μ=1 and standard deviation σ=0.5, *t*-distribution and the chi-square distributions with various degrees of freedom. The methods under comparison are the following: the Jobson and Korkie’s method [2] (JK), the Mertens’s method [21] (Mertens), the usual EL inferential method (EL), application of the delta method on the asymptotic distribution of the EL estimator of the mean and standard deviation (Delta), and the proposed method (AEL) with the adjustment level $a_{n}=0.5\log n$. Jobson and Korkie [2] assumed that the data are from a normal distribution. By applying the delta method to approximate the mean and variance of the Sharpe ratio, confidence interval for the Sharpe ratio can then be approximated by the Central Limit Theorem. Mertens [21] used the skewness and kurtosis to give an adjusted approximation of the variance of the Sharpe ratio derived in Jobson and Korkie [2] and again obtained the confidence interval of the Sharpe ratio from the Central Limit Theorem. The approach denoted by Delta is similar to JK but based on the EL. For the EL method, whenever the convex hull problem occurs for a set of simulated data, we use the convention to set the value of the profile log-EL function as negative infinity. Results are summarized in Table 1.

From Table 1, we can see that the AEL method has the most robust performance for various underlying population distributions. The AEL method always has significantly better performance over the EL method in terms of coverage probability. When the data is normally distributed, the JK method performs the best while when the data comes from a skewed distribution, the JK method performs poorly. For normal data with small sample size, the AEL has slightly less coverage probabilities than the JK method, while for normal data with sample size larger than 50 and data from various *t* distributions, the AEL has comparable performance with the JK method. For all other situations, the AEL method significantly outperforms all other methods, especially for cases with small sample sizes.

## 5. Real Data Analysis

**Figure 1:**
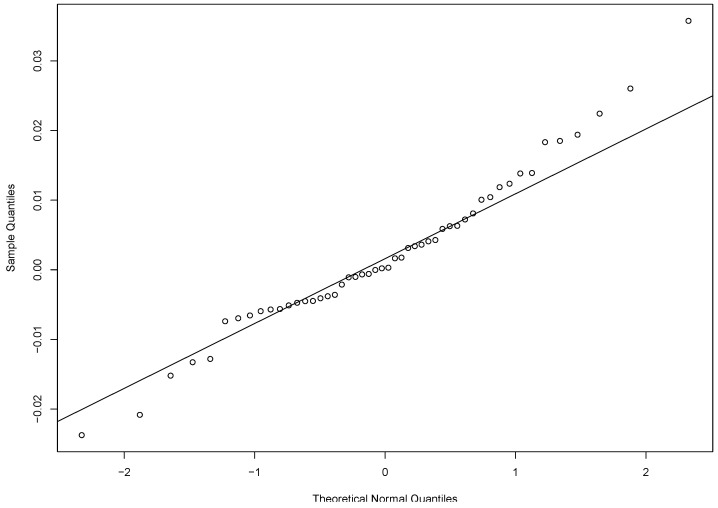
Quantile-quantile plot of Apple Inc. return data.

The data we consider is the Nasdaq GS return of the Apple Inc. (Cupertino, CA, USA) from 3 October 2017 to 12 December 2017 (https://finance.yahoo.com/quote/AAPL/). The return is evaluated from the close price of the current day compared with the close price of the previous day. There are 50 trading days during the period considered. We use the yearly return rate of the 5-year bonds, which is 2.116%, as the yearly risk-free return. Therefore, the daily risk-free return rate used in the analysis is $0.02116/252=8.397\times 10^{-5}$. Based on our data, the Durbin-Watson test statistic is 1.58. Hence, there is no significant evidence of serial correlation. The qqplot of the returns in Figure 1 reveals some skewness of the data. The confidence intervals of the Sharpe ratio for the Apple Inc. return data produced by different methods are listed in Table 2. For JK and Mertens methods, the point estimates are the value of sr that corresponding to the 50% quantile of the standard normal limiting distribution of their test statistics. The estimates of the Delta, EL and AEL methods are the value of the maximum EL and AEL estimates, respectively.

From Table 2, we see that since JK and Mertens methods are moment-based methods, both their estimates are the same as the sample Sharpe ratio. The Delta, EL and AEL methods are empirical-likelihood-based methods so the corresponding estimates are different from the previous two approaches. We observe that there is some difference in the confidence intervals for various approaches. Note that the data has some skewness as shown in Figure 1. Based on the observation from our simulation studies, the skewness will affect the JK method but not the rest of the four methods. The confidence interval based on our proposed AEL method is more robust and trustworthy.

## 6. Conclusions

We extended the adjusted empirical likelihood method [16] to obtain inference for the a parameter of interest in the presence of nuisance parameters. The advantage of the proposed method is that it does not rely on the distributional assumption of the data. In particular, we applied the proposed method to obtain inference for the Sharpe ratio. Simulation results show that the proposed method gives the coverage probabilities closest to the nominal value than those obtained by the standard empirical likelihood ratio method. Simulation results illustrate that the proposed method is comparable to Jobson and Korkie’s method [2] and outperforms the EL method when the data are from a symmetric distribution. In addition, when the data are from a skewed distribution, the proposed method outperforms all other existing methods.

The time-series properties of investment strategies can have a nontrivial impact on the Sharpe ratio estimator. In this article, we proposed using empirical-likelihood-based inference for Sharpe ratio. Empirical likelihood was motivated by independent and identically distributed data. When dealing with dependent data, we need to account for the dependency structure in constructing confidence regions for the parameter of interest. In general, the approach to handle dependent data within the EL framework is parallel to the methods based on parametric likelihood. The extension of our approach for dependent data is valuable and interesting. We will consider it in future research.

## Figures and Tables

**Table 1:** Coverage probabilities of the Sharpe ratio.

1−α	Method	n=20	n=50	n=200	n=500
N(1,0.25)					
0.9	JK	0.8956	0.9022	0.8968	0.9060
	Mertens	0.8258	0.8694	0.8894	0.9004
	EL	0.8210	0.8760	0.8906	0.9040
	AEL	0.8486	0.8874	0.8942	0.9058
	Delta	0.8428	0.8840	0.8896	0.8976
0.95	JK	0.9460	0.9488	0.9488	0.9522
	Mertens	0.8926	0.9270	0.9414	0.9494
	EL	0.8762	0.9214	0.9440	0.9514
	AEL	0.8980	0.9312	0.9466	0.9534
	Delta	0.9054	0.9334	0.9408	0.9476
$t_{3}$					
0.9	JK	0.8960	0.9004	0.9030	0.9040
	Mertens	0.8390	0.8646	0.8782	0.8890
	EL	0.8428	0.8738	0.8884	0.8946
	AEL	0.8794	0.8896	0.8944	0.8976
	Delta	0.8240	0.8586	0.8766	0.8858
0.95	JK	0.9494	0.9538	0.9516	0.9550
	Mertens	0.9028	0.9144	0.9326	0.9442
	EL	0.9042	0.9268	0.9438	0.9508
	AEL	0.9340	0.9402	0.9466	0.9514
	Delta	0.8910	0.9092	0.9318	0.9372
$t_{6}$					
0.9	JK	0.8982	0.8984	0.8934	0.8976
	Mertens	0.8738	0.8840	0.8900	0.8954
	EL	0.8700	0.8860	0.8928	0.8962
	AEL	0.8986	0.9018	0.8966	0.8978
	Delta	0.8634	0.8828	0.8936	0.9008
0.95	JK	0.9504	0.9482	0.9466	0.9476
	Mertens	0.9246	0.9364	0.9426	0.9458
	EL	0.9240	0.9394	0.9438	0.9466
	AEL	0.9466	0.9470	0.9480	0.9481
	Delta	0.9214	0.9330	0.9460	0.9494
$\chi_{4}^{2}$					
0.9	JK	0.9640	0.9532	0.9476	0.9474
	Mertens	0.8048	0.8474	0.8676	0.8938
	EL	0.7800	0.8352	0.8626	0.8942
	AEL	0.8216	0.8536	0.8664	0.8952
	Delta	0.8072	0.8354	0.8660	0.8914
0.95	JK	0.9872	0.9808	0.9808	0.9774
	Mertens	0.8780	0.9072	0.9278	0.9422
	EL	0.8562	0.8972	0.9194	0.9418
	AEL	0.8924	0.9126	0.9228	0.9430
	Delta	0.8872	0.9046	0.9252	0.9388
$\chi_{6}^{2}$					
0.9	JK	0.9466	0.9476	0.9392	0.9414
	Mertens	0.8048	0.8460	0.8760	0.8916
	EL	0.7996	0.8392	0.8728	0.8904
	AEL	0.8346	0.8568	0.8780	0.8926
	Delta	0.8236	0.8524	0.8766	0.8846
0.95	JK	0.9796	0.9776	0.9758	0.9754
	Mertens	0.8780	0.9168	0.9352	0.9412
	EL	0.8626	0.9032	0.9284	0.9402
	AEL	0.8894	0.9156	0.9326	0.9410
	Delta	0.8916	0.9170	0.9336	0.9432

**Table 2:** Confidence Intervals of the Sharpe ratio for Apple Inc. return data.

1−α	Method	Estimate	Lower Bound	Upper Bound
0.9	JK	0.1907	−0.0441	0.4254
	Mertens	0.1907	−0.0350	0.4163
	Delta	0.1926	−0.0329	0.4181
	EL	0.1926	−0.0376	0.4140
	AEL	0.1926	−0.0479	0.4241
0.95	JK	0.1907	−0.0890	0.4703
	Mertens	0.1907	−0.0783	0.4596
	Delta	0.1926	−0.0761	0.4613
	EL	0.1926	−0.0827	0.4558
	AEL	0.1926	−0.0949	0.4683

## Appendix

**Proof** **of** **Lemma** **1.** Since *M* is a symmetric positive matrix, we have C>0 and $A-BC^{-1}B^{\tau }>0$; see Theorem 16.1 in [22]. Noting that *M* has the following factorization

$$\left(\begin{array}{cc} A & B\\ B^{\tau } & C \end{array}\right)=\left(\begin{array}{cc} I & BC^{-1}\\ 0 & I \end{array}\right)\left(\begin{array}{cc} A-BC^{-1}B^{\tau } & 0\\ 0 & C \end{array}\right)\left(\begin{array}{cc} I & 0\\ {\left(BC^{-1}\right)}^{\tau } & I \end{array}\right),$$

we have

$${\left(\begin{array}{cc} A & B\\ B^{\tau } & C \end{array}\right)}^{-1}={\left(\begin{array}{cc} I & 0\\ {\left(BC^{-1}\right)}^{\tau } & I \end{array}\right)}^{-1}\left(\begin{array}{cc} {\left(A-BC^{-1}B^{\tau }\right)}^{-1} & 0\\ 0 & C^{-1} \end{array}\right){\left(\begin{array}{cc} I & BC^{-1}\\ 0 & I \end{array}\right)}^{-1}.$$

Further note that

$$\begin{array}{cc} & \left(\begin{array}{cc} 0 & 0\\ 0 & C^{-1} \end{array}\right)\\ = & {\left(\begin{array}{cc} I & 0\\ {\left(BC^{-1}\right)}^{\tau } & I \end{array}\right)}^{-1}\left(\begin{array}{cc} I & 0\\ {\left(BC^{-1}\right)}^{\tau } & I \end{array}\right)\left(\begin{array}{cc} 0 & 0\\ 0 & C^{-1} \end{array}\right)\left(\begin{array}{cc} I & BC^{-1}\\ 0 & I \end{array}\right){\left(\begin{array}{cc} I & BC^{-1}\\ 0 & I \end{array}\right)}^{-1}\\ = & {\left(\begin{array}{cc} I & 0\\ {\left(BC^{-1}\right)}^{\tau } & I \end{array}\right)}^{-1}\left(\begin{array}{cc} 0 & 0\\ 0 & C^{-1} \end{array}\right){\left(\begin{array}{cc} I & BC^{-1}\\ 0 & I \end{array}\right)}^{-1}. \end{array}$$

Above two factorizations lead to

$$\begin{array}{cc} & {\left(\begin{array}{cc} A & B\\ B^{\tau } & C \end{array}\right)}^{-1}-\left(\begin{array}{cc} 0 & 0\\ 0 & C^{-1} \end{array}\right)\\ = & {\left(\begin{array}{cc} I & 0\\ {\left(BC^{-1}\right)}^{\tau } & I \end{array}\right)}^{-1}\left[\left(\begin{array}{cc} {\left(A-BC^{-1}B^{\tau }\right)}^{-1} & 0\\ 0 & C^{-1} \end{array}\right)-\left(\begin{array}{cc} 0 & 0\\ 0 & C^{-1} \end{array}\right)\right]{\left(\begin{array}{cc} I & BC^{-1}\\ 0 & I \end{array}\right)}^{-1}\\ = & {\left(\begin{array}{cc} I & 0\\ {\left(BC^{-1}\right)}^{\tau } & I \end{array}\right)}^{-1}\left(\begin{array}{cc} {\left(A-BC^{-1}B^{\tau }\right)}^{-1} & 0\\ 0 & 0 \end{array}\right){\left(\begin{array}{cc} I & BC^{-1}\\ 0 & I \end{array}\right)}^{-1}. \end{array}$$

Since $A-BC^{-1}B^{\tau }>0$, we have ${\left(A-BC^{-1}B^{\tau }\right)}^{-1}>0$, which leads to

$${\left(\begin{array}{cc} A & B\\ B^{\tau } & C \end{array}\right)}^{-1}\geq \left(\begin{array}{cc} 0 & 0\\ 0 & C^{-1} \end{array}\right).$$

□

**Proof** **of** **Theorem** **1.** For simplicity, denote $l\left(\theta \right)=-l_{AEL}\left(\theta \right)$. Then ${\tilde{\theta }}^{\tau }={\left({\tilde{\theta }}_{1},{\tilde{\theta }}_{2}\right)}^{\tau }$ minimizes $l\left(\theta \right)=l\left(\theta_{1},\theta_{2}\right)$, and ${\tilde{\theta }}_{2}^{0}$ minimizes $l(\theta_{1}^{0},\theta_{2})$ with respect to $\theta_{2}$. Under this new notation, the test statistic becomes

$$W\left(\theta_{1}^{0}\right)=2\left[l\left(\theta_{1}^{0},{\tilde{\theta }}_{2}^{0}\right)-l\left({\tilde{\theta }}_{1},{\tilde{\theta }}_{2}\right)\right].$$

First, the following notations are needed in this proof. Let

$$Q_{1n}\left(\theta ,t\right)=\frac{1}{n+1}\sum_{i=1}^{n+1}\frac{g_{i}\left(\theta \right)}{1+t^{\tau }g_{i}\left(\theta \right)},$$

$$Q_{2n}\left(\theta ,t\right)=\frac{1}{n+1}\sum_{i=1}^{n+1}\frac{1}{1+t^{\tau }g_{i}\left(\theta \right)}{\left(\frac{\partial g_{i}\left(\theta \right)}{\partial \theta }\right)}^{\tau }t.$$

Let $\tilde{\theta }$ and $\tilde{t}=t\left(\tilde{\theta }\right)$ be the solution of

$$Q_{1n}\left(\tilde{\theta },\tilde{t}\right)=0,\;\;\;\;Q_{2n}\left(\tilde{\theta },\tilde{t}\right)=0.$$

The existence of $\tilde{\theta }$ and $\tilde{t}=t\left(\tilde{\theta }\right)$ in a neighborhood of the true parameter $\theta_{0}$ is proved in [15,16]. Note that

$$\begin{array}{cc} \frac{\partial Q_{1n}\left(\theta ,0\right)}{\partial \theta }=\frac{1}{n+1}\sum_{i=1}^{n+1}\frac{\partial g_{i}\left(\theta \right)}{\partial \theta }, & \frac{\partial Q_{1n}\left(\theta ,0\right)}{\partial t^{\tau }}=-\frac{1}{n+1}\sum_{i=1}^{n+1}g_{i}\left(\theta \right)g_{i}{\left(\theta \right)}^{\tau },\\ \frac{\partial Q_{2n}\left(\theta ,0\right)}{\partial \theta }=0, & \frac{\partial Q_{2n}\left(\theta ,0\right)}{\partial t^{\tau }}=\frac{1}{n+1}\sum_{i=1}^{n+1}{\left(\frac{\partial g_{i}\left(\theta \right)}{\partial \theta }\right)}^{\tau }. \end{array}$$

Taylor expansion of $Q_{1n}\left(\tilde{\theta },\tilde{t}\right)$ and $Q_{2n}\left(\tilde{\theta },\tilde{t}\right)$ at $\left(\theta_{0},0\right)$ gives

$$\begin{array}{cc} 0 & =Q_{1n}\left(\tilde{\theta },\tilde{t}\right)\\ & =Q_{1n}\left(\theta_{0},0\right)+\frac{\partial Q_{1n}\left(\theta_{0},0\right)}{\partial \theta }\left(\tilde{\theta }-\theta_{0}\right)+\frac{\partial Q_{1n}\left(\theta_{0},0\right)}{\partial t^{\tau }}\left(\tilde{t}-0\right)+o_{p}\left(\delta_{n}\right) \end{array}$$

$$\begin{array}{cc} 0 & =Q_{2n}\left(\tilde{\theta },\tilde{t}\right)\\ & =Q_{2n}\left(\theta_{0},0\right)+\frac{\partial Q_{2n}\left(\theta_{0},0\right)}{\partial \theta }\left(\tilde{\theta }-\theta_{0}\right)+\frac{\partial Q_{2n}\left(\theta_{0},0\right)}{\partial t^{\tau }}\left(\tilde{t}-0\right)+o_{p}\left(\delta_{n}\right), \end{array}$$

where $\delta_{n}=\left|\right|\tilde{\theta }-\theta_{0}\left|\right|+\left|\right|\tilde{t}\left|\right|$. Observing that $Q_{2n}\left(\theta_{0},0\right)=0$, we have

(A1) $$S_{n}\left(\begin{array}{c} \tilde{t}\\ \tilde{\theta }-\theta_{0} \end{array}\right)=\left(\begin{array}{c} -Q_{1n}\left(\theta_{0},0\right)+o_{p}\left(\delta_{n}\right)\\ o_{p}\left(\delta_{n}\right) \end{array}\right),$$

where

$$S_{n}={\left.\left(\begin{array}{cc} \frac{\partial Q_{1n}}{\partial t^{\tau }} & \frac{\partial Q_{1n}}{\partial \theta }\\ \frac{\partial Q_{2n}}{\partial t^{\tau }} & 0 \end{array}\right)\right|}_{\left(\theta_{0},0\right)}.$$

Now we solve (A1) for an expression of $\tilde{t}$. By the law of large numbers, as n→∞

$$\frac{1}{n}\sum_{i=1}^{n}\frac{\partial g_{i}\left(\theta \right)}{\partial \theta }⟶E\left(\frac{\partial g(\theta )}{\partial \theta }\right).$$

Therefore,

$$\frac{\partial g_{n+1}\left(\theta \right)}{\partial \theta }=-\frac{a_{n}}{n}\sum_{i=1}^{n}\frac{\partial g_{i}\left(\theta \right)}{\partial \theta }=o_{p}\left(n\right).$$

Hence applying the law of large numbers again

$$\begin{array}{cc} \frac{\partial Q_{1n}\left(\theta ,0\right)}{\partial \theta } & =\frac{1}{n+1}\sum_{i=1}^{n+1}\frac{\partial g_{i}\left(\theta \right)}{\partial \theta }\\ & =\frac{1}{n+1}\sum_{i=1}^{n}\frac{\partial g_{i}\left(\theta \right)}{\partial \theta }+\frac{1}{n+1}\frac{\partial g_{n+1}\left(\theta \right)}{\partial \theta }\\ & =E\left(\frac{\partial g(\theta )}{\partial \theta }\right)+o_{p}\left(1\right). \end{array}$$

Similarly, we can obtain

$$\frac{\partial Q_{2n}}{\partial t^{\tau }}=E{\frac{\partial g(\theta )}{\partial \theta }}^{\tau }+o_{p}\left(1\right)\;\;\;\;\;\;\;\;\mathrm{and}\;\;\;\;\;\;\;\;-\frac{\partial Q_{1n}}{\partial t^{\tau }}=Eg\left(\theta \right)g{\left(\theta \right)}^{\tau }+o_{p}\left(1\right).$$

Thus as n→∞

$$S_{n}⟶\left(\begin{array}{cc} S_{11} & S_{12}\\ S_{21} & 0 \end{array}\right)={\left.\left(\begin{array}{cc} -Egg^{\tau } & E\frac{\partial g}{\partial \theta }\\ E{\frac{\partial g}{\partial \theta }}^{\tau } & 0 \end{array}\right)\right|}_{\theta =\theta_{0}}.$$

We can see that

$$S_{n}^{-1}⟶\left(\begin{array}{cc} S_{11}^{-1}+S_{11}^{-1}S_{12}S_{22.1}^{-1}S_{21}S_{11}^{-1} & -S_{11}^{-1}S_{12}S_{22.1}^{-1}\\ -S_{22.1}^{-1}S_{21}S_{11}^{-1} & S_{22.1}^{-1} \end{array}\right),$$

where $S_{22.1}^{-1}={\left[{\left(E\frac{\partial g}{\partial \theta }\right)}^{\tau }{\left(Egg^{\tau }\right)}^{-1}\left(E\frac{\partial g}{\partial \theta }\right)\right]}^{-1}$. Consequently, (A1) can be solved as

$$\left(\begin{array}{c} \tilde{t}\\ \tilde{\theta }-\theta_{0} \end{array}\right)=S_{n}^{-1}\left(\begin{array}{c} -Q_{1n}\left(\theta_{0},0\right)+o_{p}\left(\delta_{n}\right)\\ o_{p}\left(\delta_{n}\right) \end{array}\right),$$

which means

(A2) $$\begin{array}{c} \begin{array}{cc} \tilde{\theta }-\theta_{0} & =S_{22.1}^{-1}S_{21}S_{11}^{-1}Q_{1n}\left(\theta_{0},0\right)+o_{p}\left(\delta_{n}\right)\\ \tilde{t} & =-\left(S_{11}^{-1}+S_{11}^{-1}S_{12}S_{22.1}^{-1}S_{21}S_{11}^{-1}\right)Q_{1n}\left(\theta_{0},0\right)+o_{p}\left(\delta_{n}\right). \end{array} \end{array}$$

Note that by Central Limit Theorem

$$\begin{array}{cc} Q_{1n}\left(\theta_{0},0\right) & =\frac{1}{n+1}\sum_{i=1}^{n+1}g_{i}\left(\theta_{0}\right)\\ & =\frac{n^{\frac{1}{2}}}{n+1}\cdot n^{-\frac{1}{2}}\sum_{i=1}^{n}g_{i}\left(\theta_{0}\right)-n^{-\frac{1}{2}}\cdot \frac{a_{n}}{n+1}\cdot n^{-\frac{1}{2}}\sum_{i=1}^{n}g_{i}\left(\theta_{0}\right)\\ & =n^{-\frac{1}{2}}\cdot n^{-\frac{1}{2}}\sum_{i=1}^{n}g_{i}\left(\theta_{0}\right)+o_{p}\left(n^{-\frac{1}{2}}\right), \end{array}$$

which implies

(A3) $$\sqrt{n}Q_{1n}\left(\theta_{0},0\right)⟶N\left(0,Egg^{\tau }\right)\;\;\;\;\mathrm{and}\;\;\;\;Q_{1n}=O_{p}\left(n^{-\frac{1}{2}}\right).$$

From (A2), we know that

$$\delta_{n}=\left|\right|\tilde{\theta }-\theta_{0}\left|\right|+\left|\right|\tilde{t}\left|\right|=O_{p}\left(n^{-\frac{1}{2}}\right).$$

Therefore, we have obtained the desired result

(A4) $$\tilde{t}=-\left(S_{11}^{-1}+S_{11}^{-1}S_{12}S_{22.1}^{-1}S_{21}S_{11}^{-1}\right)Q_{1n}\left(\theta_{0},0\right)+o_{p}\left(n^{-\frac{1}{2}}\right)$$

and

$$\tilde{\theta }-\theta_{0}=S_{22.1}^{-1}S_{21}S_{11}^{-1}Q_{1n}\left(\theta_{0},0\right)+o_{p}\left(n^{-\frac{1}{2}}\right).$$

In particular, we can see that

$$\tilde{t}=O_{p}\left(n^{-\frac{1}{2}}\right)\;\;\;\;\;\;\mathrm{and}\;\;\;\;\;\;\tilde{\theta }-\theta_{0}=O_{p}\left(n^{-\frac{1}{2}}\right).$$

Now we are ready to compute $l\left(\tilde{\theta }\right)=l\left({\tilde{\theta }}_{1},{\tilde{\theta }}_{2}\right)$. Taylor expansion yields

(A5) $$\begin{array}{cc} l({\tilde{\theta }}_{1},{\tilde{\theta }}_{2}) & =\sum_{i=1}^{n+1}\log\left[1+{\tilde{t}}^{\;\tau }g_{i}\left(\tilde{\theta }\right)\right]\\ & =\sum_{i=1}^{n+1}\left({\tilde{t}}^{\;\tau }g_{i}\left(\tilde{\theta }\right)-\frac{1}{2}\;{\left({\tilde{t}}^{\;\tau }g_{i}\left(\tilde{\theta }\right)\right)}^{2}\right)+o_{p}\left(1\right)\\ & ={\tilde{t}}^{\;\tau }\sum_{i=1}^{n+1}g_{i}\left(\tilde{\theta }\right)-\frac{1}{2}\;{\tilde{t}}^{\;\tau }\;\left(\sum_{i=1}^{n+1}g_{i}\left(\tilde{\theta }\right)g_{i}{\left(\tilde{\theta }\right)}^{\tau }\right)\;\tilde{t}+o_{p}\left(1\right). \end{array}$$

Note that expanding $g_{i}\left(\tilde{\theta }\right)$ at $\theta_{0}$, we get

$$g_{i}\left(\tilde{\theta }\right)=g_{i}\left(\theta_{0}\right)+\frac{\partial g_{i}\left(\theta_{0}\right)}{\partial \theta }\left(\tilde{\theta }-\theta_{0}\right)+O_{p}\left(n^{-1}\right),$$

for i=1,2,…,n. Hence

$$\begin{array}{cc} \sum_{i=1}^{n}g_{i}\left(\tilde{\theta }\right) & =\sum_{i=1}^{n}g_{i}\left(\theta_{0}\right)+\sum_{i=1}^{n}\frac{\partial g_{i}\left(\theta_{0}\right)}{\partial \theta }\cdot \left(\tilde{\theta }-\theta_{0}\right)+O_{p}\left(1\right)\\ & =nQ_{1n}\left(\theta_{0},0\right)+nS_{12}S_{22.1}^{-1}S_{21}S_{11}^{-1}Q_{1n}\left(\theta_{0},0\right)+o_{p}\left(n^{\frac{1}{2}}\right) \end{array}$$

and

$$g_{n+1}\left(\tilde{\theta }\right)=-\frac{a_{n}}{n}\sum_{i=1}^{n}g_{i}\left(\tilde{\theta }\right)=o_{p}\left(n^{\frac{1}{2}}\right).$$

Consequently, we can obtain the first term of (A5) as

$${\tilde{t}}^{\;\tau }\sum_{i=1}^{n+1}g_{i}\left(\tilde{\theta }\right)=-n\;Q_{1n}{\left(\theta_{0},0\right)}^{\tau }\left(S_{11}^{-1}+S_{11}^{-1}S_{12}S_{22.1}^{-1}S_{21}S_{11}^{-1}\right)Q_{1n}\left(\theta_{0},0\right)+o_{p}\left(1\right).$$

Now we calculate the second term of (A5). For i=1,2,…,n,

$$g_{i}\left(\tilde{\theta }\right)g_{i}{\left(\tilde{\theta }\right)}^{\tau }=g_{i}\left(\theta_{0}\right)g_{i}{\left(\theta_{0}\right)}^{\tau }+O_{p}\left(n^{-\frac{1}{2}}\right).$$

Thus

$$\Sigma_{i=1}^{n}g_{i}\left(\tilde{\theta }\right)g_{i}{\left(\tilde{\theta }\right)}^{\tau }=\sum_{i=1}^{n}g_{i}\left(\theta_{0}\right)g_{i}{\left(\theta_{0}\right)}^{\tau }+O_{p}\left(n^{\frac{1}{2}}\right)=-nS_{11}+O_{p}\left(n^{\frac{1}{2}}\right).$$

Note that

$$g_{n+1}\left(\tilde{\theta }\right)g_{n+1}{\left(\tilde{\theta }\right)}^{\tau }=o_{p}\left(n^{\frac{1}{2}}\right)o_{p}\left(n^{\frac{1}{2}}\right)=o_{p}\left(n\right).$$

We have

$${\tilde{t}}^{\;\;\tau }\;\left(\sum_{i=1}^{n+1}g_{i}\left(\tilde{\theta }\right)g_{i}{\left(\tilde{\theta }\right)}^{\tau }\right)\;\tilde{t}=-n\;Q_{1n}{\left(\theta_{0},0\right)}^{\tau }\left(S_{11}^{-1}+S_{11}^{-1}S_{12}S_{22.1}^{-1}S_{21}S_{11}^{-1}\right)Q_{1n}\left(\theta_{0},0\right)+o_{p}\left(1\right).$$

Finally, we have

(A6) $$l\left({\tilde{\theta }}_{1},{\tilde{\theta }}_{2}\right)=-\frac{n}{2}Q_{1n}{\left(\theta_{0},0\right)}^{\tau }\left(S_{11}^{-1}+S_{11}^{-1}S_{12}S_{22.1}^{-1}S_{21}S_{11}^{-1}\right)Q_{1n}\left(\theta_{0},0\right)+o_{p}\left(1\right).$$

Similarly, we can apply the above process to $l(\theta_{1}^{0},{\tilde{\theta }}_{2}^{0})$. The procedures are sketched as follows. Let ${\tilde{\theta }}_{2}^{0}$ and $\tilde{t_{0}}=t\left(\theta_{1}^{0},{\tilde{\theta }}_{2}^{0}\right)$ satisfy

$$Q_{1n}\left(\theta_{1}^{0},{\tilde{\theta }}_{2}^{0},\tilde{t_{0}}\right)=0\;\;\;\;\;\;\mathrm{and}\;\;\;\;\;\;Q_{2n}\left(\theta_{1}^{0},{\tilde{\theta }}_{2}^{0},\tilde{t_{0}}\right)=0.$$

Expanding $Q_{1n}$ and $Q_{2n}$ at $\left(\theta_{1}^{0},\theta_{2}^{0},0\right)$ will produce the linear equations

(A7) $$H_{n}\left(\begin{array}{c} \tilde{t_{0}}\\ {\tilde{\theta }}_{2}^{0}-\theta_{2}^{0} \end{array}\right)=\left(\begin{array}{c} -Q_{1n}\left(\theta_{0},0\right)+o_{p}\left(\delta_{n}^{\prime }\right)\\ o_{p}\left(\delta_{n}^{\prime }\right) \end{array}\right),$$

where $\theta_{0}=\left(\theta_{1}^{0},\theta_{2}^{0}\right)$ is the true value of θ, $\delta_{n}^{\prime }=\left|\right|{\tilde{\theta }}_{2}^{0}-\theta_{2}^{0}\left|\right|+\left|\right|\tilde{t_{0}}\left|\right|$ and as n→∞

$$H_{n}⟶\left(\begin{array}{cc} H_{11} & H_{12}\\ H_{21} & 0 \end{array}\right)={\left.\left(\begin{array}{cc} -Egg^{\tau } & E\frac{\partial g}{\partial \theta_{2}}\\ E{\frac{\partial g}{\partial \theta_{2}}}^{\tau } & 0 \end{array}\right)\right|}_{\theta =\theta_{0}}.$$

Note that $H_{11}=S_{11}$. Solving (A7) gives us

(A8) $$\tilde{t_{0}}=-\left(H_{11}^{-1}+H_{11}^{-1}H_{12}H_{22.1}^{-1}H_{21}H_{11}^{-1}\right)Q_{1n}\left(\theta_{0},0\right)+o_{p}\left(n^{-\frac{1}{2}}\right)$$

and

$${\tilde{\theta }}_{2}^{0}-\theta_{2}^{0}=H_{22.1}^{-1}H_{21}H_{11}^{-1}Q_{1n}\left(\theta_{0},0\right)+o_{p}\left(n^{-\frac{1}{2}}\right).$$

By Taylor expansion, the above estimations yield

(A9) $$l\left(\theta_{1}^{0},{\tilde{\theta }}_{2}^{0}\right)=-\frac{1}{2}nQ_{1n}{\left(\theta_{0},0\right)}^{\tau }\left(H_{11}^{-1}+H_{11}^{-1}H_{12}H_{22.1}^{-1}H_{21}H_{11}^{-1}\right)Q_{1n}\left(\theta_{0},0\right)+o_{p}\left(1\right).$$

Using (A9) and (A6), we can write

$$\begin{array}{cc} W(\theta_{1}^{0})= & 2l\left(\theta_{1}^{0},{\tilde{\theta }}_{2}^{0}\right)-2l\left({\tilde{\theta }}_{1},{\tilde{\theta }}_{2}\right)\\ = & {\left[{\left(Egg^{\tau }\right)}^{-\frac{1}{2}}\sqrt{n}Q_{1n}\left(\theta_{0},0\right)\right]}^{\tau }\left(A-B\right)\left[{\left(Egg^{\tau }\right)}^{-\frac{1}{2}}\sqrt{n}Q_{1n}\left(\theta_{0},0\right)\right]+o_{p}\left(1\right), \end{array}$$

where

$$A={\left(Egg^{\tau }\right)}^{-\frac{1}{2}}\left(E\frac{\partial g}{\partial \theta }\right){\left[{\left(E\frac{\partial g}{\partial \theta }\right)}^{\tau }{\left(Egg^{\tau }\right)}^{-1}\left(E\frac{\partial g}{\partial \theta }\right)\right]}^{-1}{\left(E\frac{\partial g}{\partial \theta }\right)}^{\tau }{\left(Egg^{\tau }\right)}^{-\frac{1}{2}}$$

$$B={\left(Egg^{\tau }\right)}^{-\frac{1}{2}}\left(E\frac{\partial g}{\partial \theta_{2}}\right){\left[{\left(E\frac{\partial g}{\partial \theta_{2}}\right)}^{\tau }{\left(Egg^{\tau }\right)}^{-1}\left(E\frac{\partial g}{\partial \theta_{2}}\right)\right]}^{-1}{\left(E\frac{\partial g}{\partial \theta_{2}}\right)}^{\tau }{\left(Egg^{\tau }\right)}^{-\frac{1}{2}}$$

and all the evaluations related to *g* are performed at the true value $\theta_{0}$. By assumption, $E\frac{\partial g}{\partial \theta }$ has rank *k* and $Egg^{\tau }$ is positive definite. Therefore, both *A* and *B* are non-negative definite and idempotent. By Lemma 1

$$\begin{array}{cc} & \left(E\frac{\partial g}{\partial \theta }\right){\left[{\left(E\frac{\partial g}{\partial \theta }\right)}^{\tau }{\left(Egg^{\tau }\right)}^{-1}\left(E\frac{\partial g}{\partial \theta }\right)\right]}^{-1}{\left(E\frac{\partial g}{\partial \theta }\right)}^{\tau }\\ \geq & \left(E\frac{\partial g}{\partial \theta_{1}},E\frac{\partial g}{\partial \theta_{2}}\right)\left(\begin{array}{cc} 0 & 0\\ 0 & {\left[{\left(E\frac{\partial g}{\partial \theta_{2}}\right)}^{\tau }{\left(Egg^{\tau }\right)}^{-1}\left(E\frac{\partial g}{\partial \theta_{2}}\right)\right]}^{-1} \end{array}\right)\left(\begin{array}{c} E{\frac{\partial g}{\partial \theta_{1}}}^{\tau }\\ E{\frac{\partial g}{\partial \theta_{2}}}^{\tau } \end{array}\right)\\ = & \left(E\frac{\partial g}{\partial \theta_{2}}\right){\left[{\left(E\frac{\partial g}{\partial \theta_{2}}\right)}^{\tau }{\left(Egg^{\tau }\right)}^{-1}\left(E\frac{\partial g}{\partial \theta_{2}}\right)\right]}^{-1}{\left(E\frac{\partial g}{\partial \theta_{2}}\right)}^{\tau }, \end{array}$$

which means that A−B is non-negative definite. Thus by Result 2, A−B is also idempotent. From (A3), we can see that ${\left(Egg^{\tau }\right)}^{-\frac{1}{2}}\sqrt{n}Q_{1n}\left(\theta_{0},0\right)$ follows the multivariate standard normal distribution asymptotically. Note that tr(A)=k and tr(B)=k−q. We have tr(A−B)=k−(k−q)=q. The requirement of Lemma 1 is satisfied, which implies

$$W\left(\theta_{1}^{0}\right)\overset{\;d\;}{\to }\chi_{q}^{2}.$$

□
